# Floral nectar production and carbohydrate composition and the structure of receptacular nectaries in the invasive plant *Bunias orientalis* L. (Brassicaceae)

**DOI:** 10.1007/s00709-015-0902-6

**Published:** 2015-11-11

**Authors:** Bożena Denisow, Marzena Masierowska, Sebastian Antoń

**Affiliations:** Uniwersytet Przyrodniczy w Lublinie, Lublin, Poland

**Keywords:** *Bunias orientalis*, Nectar secretion and composition, Receptacular nectaries, Anatomy and ultrastructure

## Abstract

The data relating to the nectaries and nectar secretion in invasive Brassicacean taxa are scarce. In the present paper, the nectar production and nectar carbohydrate composition as well as the morphology, anatomy and ultrastructure of the floral nectaries in *Bunias orientalis* were investigated. Nectary glands were examined using light, fluorescence, scanning electron and transmission electron microscopy. The quantities of nectar produced by flowers and total sugar mass in nectar were relatively low. Total nectar carbohydrate production per 10 flowers averaged 0.3 mg. Nectar contained exclusively glucose (G) and fructose (F) with overall G/F ratio greater than 1. The flowers of *B. orientalis* have four nectaries placed at the base of the ovary. The nectarium is intermediate between two nectary types: the lateral and median nectary type (lateral and median glands stay separated) and the annular nectary type (both nectaries are united into one). Both pairs of glands represent photosynthetic type and consist of epidermis and glandular tissue. However, they differ in their shape, size, secretory activity, dimensions of epidermal and parenchyma cells, thickness of secretory parenchyma, phloem supply, presence of modified stomata and cuticle ornamentation. The cells of nectaries contain dense cytoplasm, plastids with starch grains and numerous mitochondria. Companion cells of phloem lack cell wall ingrowths. The ultrastructure of secretory cells indicates an eccrine mechanism of secretion. Nectar is exuded throughout modified stomata.

## Introduction

Nectar production is generally associated with mutualistic relations with animals that rely on sugar secretions in their nutrition (Simpson and Neff 1981; Nepi et al. 2009). Indeed, nectaries attract pollinators, protectors (e.g. ants and parasitoids) against herbivores or even prey in carnivorous plants (Heil 2011). These structures occur in different plant organs. Glands located in the flowers are commonly designated as floral nectaries, whereas extrafloral nectaries are present in other parts of the plant.

Location, morphology and structure of the floral nectaries have been described for numerous plant species, and nectary traits are considered highly diverse in angiosperms since those structures appeared independently in many taxa (Vogel 1997; Fahn 2000). Moreover, the relative location of nectaries within a flower is under pressure to maximize relations with pollinators (Nepi 2007) and hence to ensure the deposition of pollen on the stigma by pollinators.

In the family Brassicaceae, both floral (Deng and Hu 1995; Davis et al. 1986, 1998) and extrafloral (Mathur et al. 2013) nectaries have been reported. Floral nectaries are outgrowths of the receptacle located between the sepals and stamens in the region of filament bases (Frei 1955; Fahn 1979a). As a result, floral insect visitors collecting nectar often inadvertently pick up and disperse pollen. Floral nectaries of crucifers vary in their morphology, size and distribution. There are four nectary types based on number and distribution of the organs: (1) annular—a continuous zone of nectarial tissue around the receptacle, e.g. *Dentaria* sp. and *Sisymbrium loeselii*; (2) two-nectary type—two opposing nectaries at the flower base, e.g. *Hesperis matronalis* and *Matthiola bicornis*; (3) four-nectary type—made up of two pairs of nectaries classified as lateral (associated with the short stamens) and median (external to the long stamens) as in *Brassica*, *Sinapis* and *Raphanus*; and (4) eight-nectary type—two pairs of lateral and two pairs of median nectaries, e.g. *Lobularia maritima* (Norris 1941, cit. in Davis et al. 1996; Clemente Muñoz and Hernández Bermejo 1978; Davis et al. 1998). The pairs of lateral and median nectaries vary with respect to their secretory activity as well as vascularization (Davis et al. 1986, 1998). In general, the lateral nectaries produce most of a flower’s nectar and total nectar carbohydrates (Eisikowitch 1981; Davis et al. 1986, 1996, 1998; Masierowska 2003). Brassicaceae nectaries are composed of three types of tissues: (1) epidermal tissue, (2) parenchyma and (3) vascular tissue. The vascular tissue is composed of phloem alone (e.g. Frei 1955, Fahn 1979a, Davis et al. 1986, 1998). Modified stomata are the way of nectar exudation (Davis et al. 1998, Masierowska 2003).

According to Fahn (2000), the origin of secreted nectar is the phloem sap. However, nectar carbohydrates may also be directly derived from photosynthesis by nectary parenchyma cells (Pacini et al. 2003). As reported by Lüttge (2013), the photosynthetic capacity of green nectaries is of the same order of magnitude as that of leaves, and photosynthesis in these glands can potentially provide sugars and reduction equivalents for the redox cycle of nectar. Green nectaries occur in many plant species. Also, floral nectaries in the Brassicaceae consist of parenchyma with chloroplasts (Davis et al. 1986). The origin of nectar carbohydrates can impact on nectar volume, carbohydrate concentration and composition, which directly influence the activity and type of floral insect visitors, and thus play an important role in the reproductive success of a flower (Heil 2011).

Although the nectaries and attractiveness of floral nectar to potential pollinators in many crucifers, including economically important species, have been extensively studied (e.g. Pierre et al. 1999; Kołtowski 2002; Masierowska 2003; Denisow 2005, Masierowska and Piętka 2014), the data on the nectary structure and nectar production in invasive Brassicacean taxa are scarce.

One of very aggressive invaders is the Turkish cabbage, *Bunias orientalis* L. (Brassicaceae: tribe Buniadeae). It is believed that this species bears a threat to native flora due to its high colonization rates and ability to form dominant stands (Dietz et al. 1999; Hochkirch et al. 2012). Nevertheless, the presence of introduced plants may be beneficial (Bjerknes et al. 2007; Bartomeus et al. 2008) as they may contribute to a higher food diversity and thus increase pollinator species richness or abundance (Hochkirch et al. 2012). According to Schürkens and Chittka (2001), nectar production in *B. orientalis* flowers is relatively low, but as a result of extremely high flower display, this species may be a valuable food source for visiting insects and hence compete for pollinators with native flora and depress the pollinator visits to other plants (Hochkirch et al. 2012).

Understanding various reproductive traits of invasive species may contribute to the design of eradication or control programmes. The reproductive success, i.e. seed setting, in the alien species often depends on the activity of the flower visitors, which is affected also by nectar availability. Thus, providing information about nectar production in the invader flowers might be useful in estimation of chances of controlling its spread. For now, a description of *B. orientalis* nectaries is incomplete and data on floral nectar characteristics are lacking. Our aims are to examine (i) the anatomy and ultrastructure of the floral nectaries and (ii) nectar production and carbohydrate composition, with emphasis on possible changes in nectar composition between individual plants. The present study is performed in the framework of a research programme on the reproductive biology of *B. orientalis* in the invaded areas.

## Material and methods

### Study plant and area

Turkish cabbage, *B. orientalis* L., is a biennial or perennial hemicryptophyte with allelopathic ability that enables rapid dominance and promotes the formation of dense patches. The numerous little flowers (petals are 5–8 mm long) are hermaphroditic and insect-pollinated, with self-fertile breeding system (Dietz et al. 1999). Plants reach up to 1.2 m height.

During the last decades, *B. orientalis* has invaded large areas in North America and Europe (Doll 2005; Laivins et al. 2006). Natural habitats of this species include woodland, sunny edges of forests, dappled shade and riverbanks. In its new range, *B. orientalis* occurs in dry and sunny areas, on roadsides, adjacent grasslands, slopes and invades predominantly disturbed habitats (Birnbaum 2006).

Plants of *B. orientalis*, growing in a dense patch in Lublin (51°08′–51° 18′ N, 22°27′–22° 41′ E), SE Poland, were studied in the field and sampled during 2007, 2009 and 2012. The experimental patch (10 m × 20 m) was covered by the stable phytocoenoses the *Bunietum orientalis* developed on a sandy-dusty, grey-brown soil (pH in 1 n KCl = 7.4).

### Nectar production

Nectar production was studied during a peak of flowering, i.e. in late May. In order to assess flower longevity and period of nectar production, flower buds (*n* = 20 per year) were randomly chosen and tagged just before opening and their development and presence of nectar were observed. Nectar production was investigated by covering whole plants (*n* = 10–15 per year) that had flowers in the bud stage, with tulle isolators (mesh size < 1 mm). Plants were kept bagged until nectar sampling. Two-day flowers were collected from isolated plants between 09:00 and 10:00 and transported immediately into the laboratory. Then, the nectar amount (in mg) produced in an individual flower was measured using Jabłoński’s pippetes (Jabłoński 2002) and a WPS-36 analytical balance (RADWAG, Radom, Poland). Each year of a study, nectar samples were taken on three dates in 3–5 replications. A single sample contained nectar collected from 5 to 10 flowers. Nectar sugar concentration (% wt/ total wt) was measured using a RL-4 refractometer (PZO, Warszawa Poland). Then, nectar amount and sugar concentration of nectar were used to calculate the total sugar amount (in mg) secreted in nectar per 10 flowers.

### Nectar carbohydrate analysis

Nectar carbohydrate composition was analysed in spring 2012. A total of 15 nectar samples were taken from unvisited flowers randomly chosen on five different plant individuals—three samples per individual. Each sample contained nectar from 10 to 15 flowers. Nectar was collected using wicks (8 × 15 mm) of Whatman No. 1 filter paper (McKenna and Thomson 1988). Next, the nectar-laden wicks were pinned, air-dried and stored at −20 °C. Before analysis, wicks were thawed to room temperature. Then, to recover nectar from the filter paper, they were placed in 0.6 ml distilled water in Eppendorf centrifuge tubes and spun for 0.5 h at 10,000 (rpm) at 15 °C. The analysis of nectar carbohydrates was carried out using the HPLC technique. Following filtration of the sample using a sterile syringe filter (0.45 μm pore size), 0.3 ml per sample was analysed by a Gilson HPLC apparatus equipped with Knauer’s RI *K-*2300 refractometric detector. Carbohydrates were separated using an Aminex HPX - 87H column (300 mm long, 7.8 mm i.d.) coupled with a Bio-Rad guard column (30 mm long, 4.6 mm i.d.). Elution of carbohydrates was carried out using a mobile phase comprising 30 mM sulphuric acid (0.5 ml min^−1^) at a column temperature of 42 °C. The contents of fructose, glucose and sucrose were determined and expressed as a percentage of total sugars.

### Nectary structure

In 2012, the structure of floral nectaries was studied in nectar-bearing flowers at the 2nd day of anthesis. The distribution of secretory glands in fresh flowers (*n* = 10) was investigated under an Olympus SZX12 stereoscopic microscope. Then, the nectaries were prepared for investigations by means of light microscopy (LM), transmission electron microscopy (TEM) and scanning electron microscopy (SEM).

For LM examination, semi-thin sections of nectaries were prepared. Floral material was fixed in 2.5 % glutaraldehyde in phosphate buffer (pH 7.4; 0.1 M) for 12 h at 4 °C, followed by three washes in phosphate buffer. Then, it was treated with 1 % osmium tetraoxide solution at 0 °C for 1.5 h and washed three times in distilled water. After dehydration in a graded ethanol series, the samples were infiltrated in a medium grade LR White acrylic resin (Sigma-Aldrich). Following polymerization at 60 °C, sections were cut by a Reichert Ultracut-S ultramicrotome and a glass knife at a thickness of 0.7–0.9 μm. For general histology, semi-thin sections were stained with 1 % (*w*/*v*) aqueous methylene blue-Azur B solution. The presence of insoluble polysaccharides was tested with Periodic acid-Schiff’s (PAS) reagent after blocking free aldehyde groups (O’Brien and McCully 1981). LM observations were conducted by means of a Nikon Eclipse E200 (Nikon Corp., Tokyo, Japan), and measurements were taken with NIS-Elements Br 2 imaging software (Nikon Corp., Tokyo, Japan).

Moreover, other semi-thin sections were stained with auramine O for the presence of cutinized cell walls (Heslop-Harrison 1977) and examined by means of a Nikon Eclipse 90i equipped with fluorescein isothiocyanate filter (EXP. 465-495, DM 505; BA 515-555). Autofluorescence of chlorophyll in plastids was tested in fresh, hand-cut sections of nectary using a Nikon 90i fluorescence microscope with UV-2B filter. In each case, control sections were used.

The material for TEM was fixed as above. Ultra-thin sections (60–70 nm thick) were cut from the embedded material, subsequently stained with uranyl acetate and post-stained in lead citrate (Reynolds 1963). Then, the sections were examined with an FEI Technai-G2 Spirit Bio TWIN transmission electron microscope at an accelerating voltage of 120 kV. TEM images were taken using a Megaview G2 Olympus Soft Imaging Solution camera.

For observations in SEM, flower bases were fixed in 2.5 % glutaraldehyde in phosphate buffer (pH 7.4; 0.1 M) at 4 °C for 12 h. The material was then washed in phosphate buffer and dehydrated in a graded acetone series, respectively. The plant material was subsequently subjected to critical-point drying using liquid CO_2_, sputter-coated with gold and examined with TESCAN/VEGA LMU SEM (TESCAN, Brno, Czech Republic) at an accelerating voltage of 30 kV.

### Data analysis

Descriptive statistics were calculated for data on nectar characteristics and nectar tissues measurements and are presented as mean values ± SD (standard deviation). For glucose, fructose and sucrose content in nectar, coefficient of variation (CV) was computed. The differences in nectar amount, nectar concentration and nectar sugar quantity per flower between years of study were subjected to separate one-way ANOVAs. Additionally, the differences in the mean values of nectar carbohydrate composition (glucose and fructose) between individual plants were analysed. When significant differences were detected, post hoc comparison was made by means of the HSD Tukey test. The level of statistical significance required to measure differences between the means for all analyses was *P* = 0.05. All data analyses were performed using STATISTICA 6.0 (StatSoft Inc., Kraków, Poland) software.

## Results

### Nectar production and composition

**Fig. 1:**
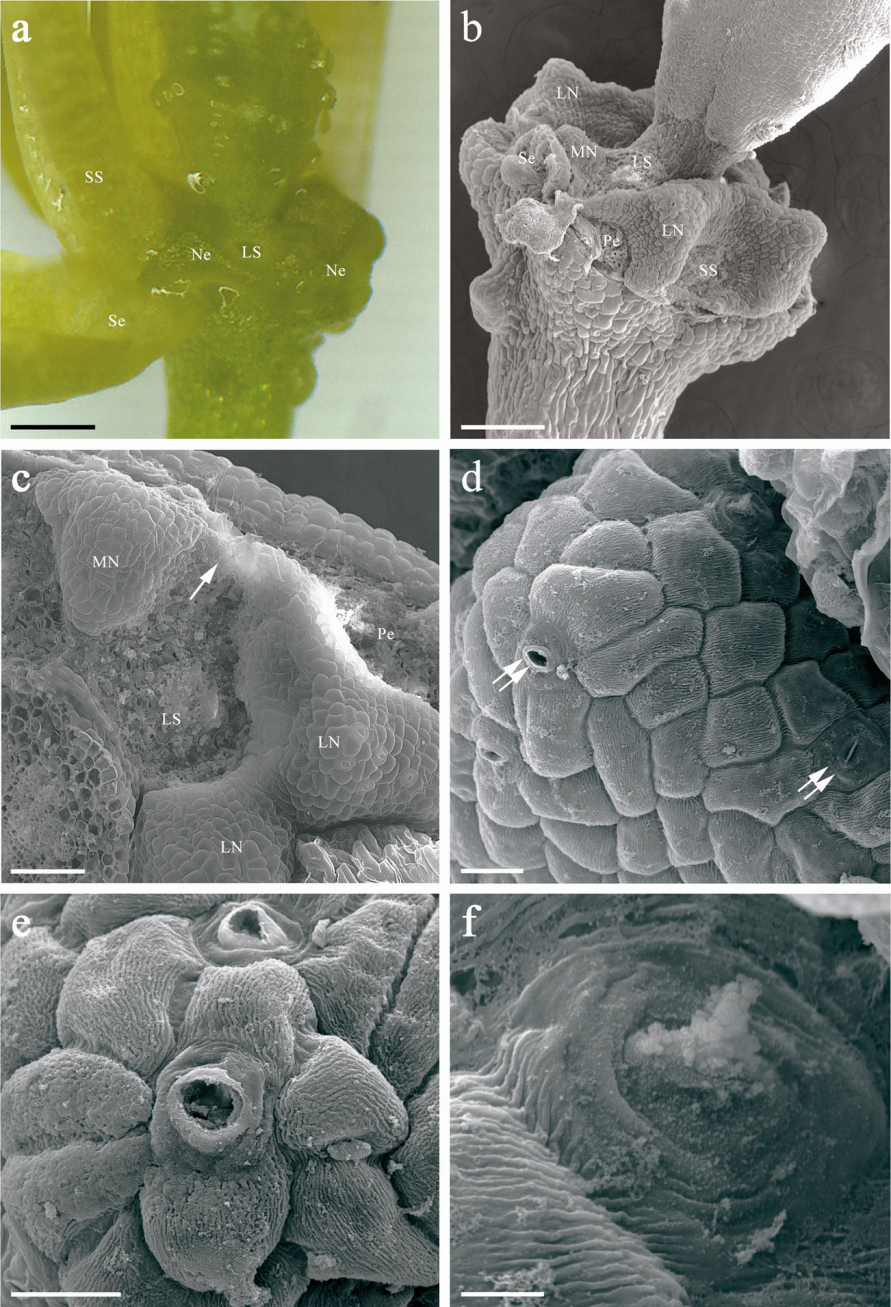
View of the flower (**a**) and scanning electron micrographs (**b**–**f**) of the floral nectaries of *Bunias orientalis*: **a** Flower devoid of part of perianth and stamens, showing the position of a green nectarium. Note nectar glistening on the nectarium surface. *Scale bar* = 500 μm. **b** Floral base after removal of perianth and stamens with lateral and median nectaries. Lateral nectary encircles short stamen. *Scale bar* = 200 μm. **c** Floral nectaries from above; note a narrow band of nectarial tissue (*arrow*) connecting lateral nectary to adjacent median one. *Scale bar* = 100 μm. **d**–**e** Mature and immature (far right of Fig. 1d) modified stomata (*double arrows*) placed on the same level as other epidermal cells. Note distinctly striated surface of nectary epidermis. *Scale bars* = 20 μm. **f** Mature stoma demonstrating pore occlusion. *Scale bar* = 5 μm

Life span of a free-pollinated *B. orientalis* flower was approximately 1.8 to 3.2 days (2.2 days on average). Nectar release began with the opening of the flower, along with the start of pollen presentation (i.e. when at least one anther opened) and lasted to the end of anthesis (i.e. when perianth began to wilt). The floral nectar was produced by nectaries situated adjacent to the stamen filament bases and was accumulated between filaments (Fig. 1a).

The data concerning nectar production are presented in Table 1. The amount of nectar produced per 10 flowers varied widely from 0.2 to 3.7 mg and averaged 1.1 mg. Statistical analysis showed that the nectar amount demonstrated a significant year effect (*F*_2, 30_ = 40.489, *P* < 0.001). In 2009, flowers of investigated species produced 7.8× and 3.4× more nectar than in 2007 and 2012, respectively. The nectar concentration was moderate and ranged between 12.0 and 36.0 %. No significant year effect on the nectar sugar concentration (*F*_2, 30_ = 0.418, *P* = 0.665) was detected. The total mass of sugar in nectar per 10 flowers differed significantly between the years of study (*F*_2, 30_ = 48.796, *P* = 0.001). The relationship was similar to that stated for the nectar quantity. The highest mean total mass of sugar in nectar was produced in 2009 and reached 0.9 mg/10 flowers. These differences were caused by weather conditions. Hotter temperatures and shortage of precipitation in 2007 and 2012, as compared with 2009, impaired nectar yield and sugar production.

**Table 1:** Nectar amount (mg), sugar concentration (% wt/total wt) and total sugar mass secreted in nectar (mg) per 10 flowers of *Bunias orientalis* during a 3 year study. Values are mean ± SD

Year	Nectar amount	Sugar concentration of nectar	Total sugar mass in nectar
2007	0.4a ± 0.2	27.5a ± 3.8	0.1a ± 0.1
2009	3.1b ± 0.9	31.2a ± 0.5	0.9b ± 0.3
2012	0.9c ± 0.4	29.1a ± 7.6	0.3c ± 0.2
Mean	1.1 ± 0.7	28.5 ± 5.5	0.3 ± 0.2

Means within columns followed by the same letter do not significantly differ at *P* < 0.05, based on HSD Tukey test

The nectar contained exclusively glucose (G) and fructose (F), and no other carbohydrates were detected. The complete absence of sucrose was characteristic of the floral nectar of *B. orientalis*. On average, the nectar contained 58.8 ± 4.1 % of glucose and 41.2 ± 4.1 % of fructose in the nectar profile (Table 2). Glucose always exceeded fructose with ratios above 1. Statistical comparison showed significant differences between individual plants both for glucose and fructose content in nectar (*F*_4, 10_ = 13.273, *P* < 0.001) as well for G/F ratio (*F*_4, 10_ = 11.563, *P* < 0.001).

**Table 2:** Percentage nectar carbohydrate composition and glucose: fructose ratios among individuals of *Bunias orientalis* from five plants sampled in 2012

Plant	Glucose (G)		Fructose (F)		G/F	
	Mean ± SD	CV	Mean ± SD	CV	Mean ± SD	CV
1 (*n* = 3)	53.3a ± 2.0	(3.7)	46.7a ± 2.0	(4.3)	1.1a ± 0.1	(9.1)
2 (*n* = 3)	56.6ab ± 2.4	(4.2)	43.4ab ± 2.4	(5.5)	1.3ab ± 0.1	(7.7)
3 (*n* = 3)	59.2bc ± 0.8	(1.3)	40.8bc ± 0.8	(1.9)	1.5abc ± 0.1	(6.6)
4 (*n* = 3)	61.1bc ± 1.0	(1.6)	38.9bc ± 1.0	(2.6)	1.6bc ± 0.1	(6.3)
5 (*n* = 3)	63.7c ± 2.6	(4.1)	36.3c ± 2.6	(7.2)	1.8c ± 0.2	(11.1)
Mean	58.8 ± 4.1	(6.9)	41.2 ± 4.1	(9.9)	1.4 ± 0.2	(14.3)

Means within the columns with the same letter do not differ significantly at *P* < 0.05, based on HSD Tukey test

*CV* coefficient of variation in %

### Nectary structure

**Fig. 2:**
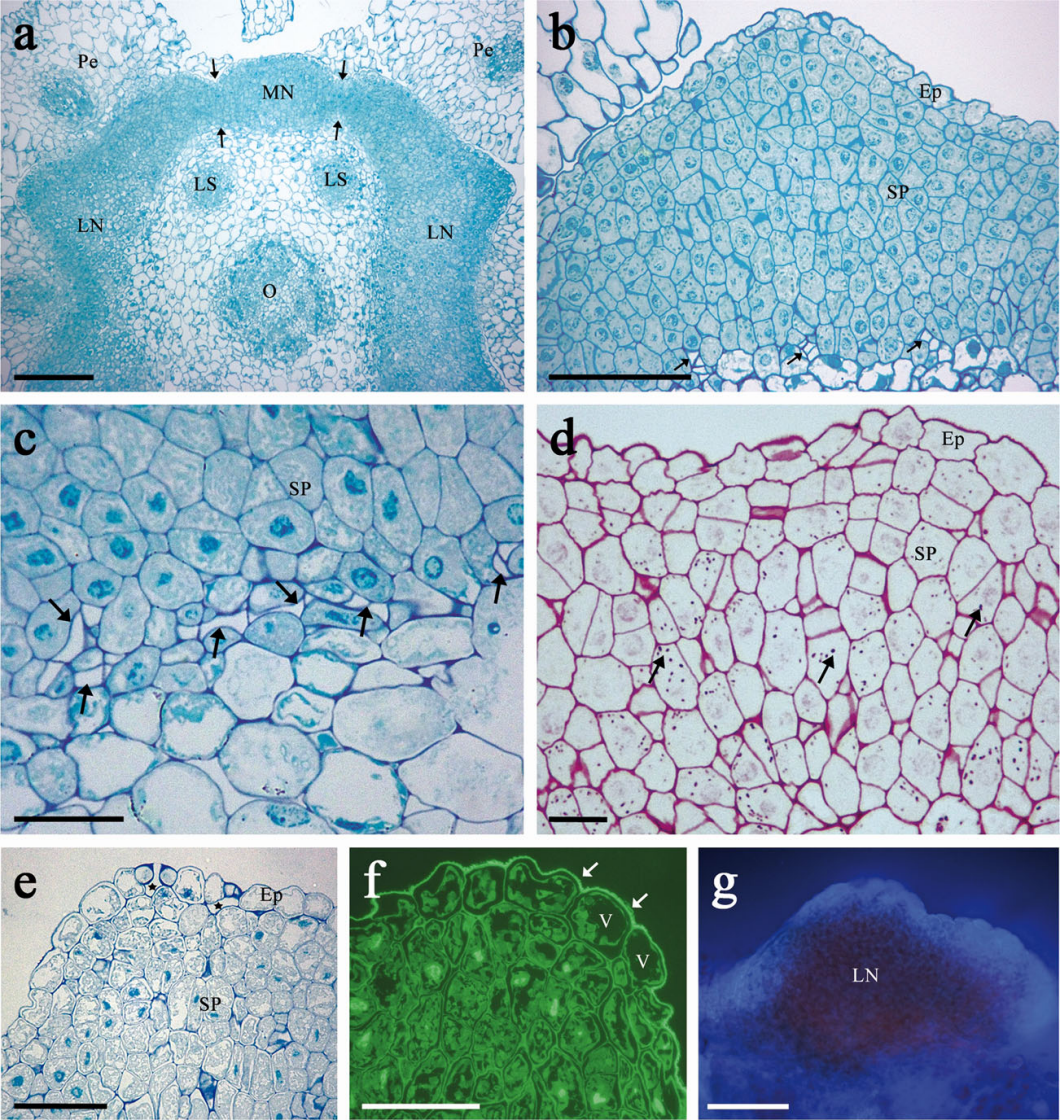
Light micrographs of sections through the lateral nectary of *B. orientalis*: **a** Confluence of lateral and median nectaries at their bases (*arrows*) in a flower sectioned transversely and then stained with methylene blue-Azur B. *Scale bar* = 200 μm. **b** Section of nectary showing intensely stained cytoplasm of epidermis and secretory parenchyma cells, and phloem strands (*arrows*) at the base of the secretory gland; staining with methylene blue-Azur B. *Scale bar* = 100 μm. **c** Sieve tube elements (*arrows*) next to the base of nectary; staining with methylene blue-Azur B. *Scale bar* = 50 μm. **d** Staining with PAS revealing the presence of numerous small starch grains (*arrows*) in secretory parenchyma cells, mainly found in the deeper layers of this tissue. *Scale bar* = 20 μm. **e** Nectary epidermis with open modified stomata, note substomatal spaces (*asterisks*). Longitudinal section stained with methylene blue-Azur B. The stoma on the right is sectioned obliquely. *Scale bar* = 50 μm. **f** Cuticle on the surface of nectary epidermis fluoresces with auramine O. *Scale bar* = 50 μm. **g** Autofluorescence of chlorophyll in subepidermal parenchyma when exposed to UV light. *Scale bar* = 100 μm

The flowers of *B. orientalis* have four green nectaries (two lateral and two median) placed at the base of the ovary (Fig. 1a–b). Lateral nectaries are surrounded by the filaments of two petals and one short stamen, while medians are located outside the filaments of two long stamens. Narrow bands of nectarial tissue connecting lateral nectaries to adjacent median ones were observed (Figs. 1c and 2a). As a result, medians were often confluent with laterals. Both types of the nectaries differed in terms of their morphology and anatomy (Table 3).

**Table 3:** Characteristics of two types of nectaries in *Bunias orientalis* flowers. Values are mean ± SD, *n* = 15 for each examined feature

Parameter	Lateral nectary	Median nectary
Dimensions of epidermal cells (μm)	11.7 × 17.0	14.1 × 16.0
Thickness of the epidermis layer with cuticle (μm)	11.7 ± 2.3	14.1 ± 2.1
Thickness of the outer cell wall of epidermal cells (μm)	0.8 ± 0.2	0.9 ± 0.2
Thickness of the secretory parenchyma layer (μm)	138.4 ± 28.2	121.2 ± 15.5
Dimensions of the secretory parenchyma cells (μm)	15.5 × 13.3	14.1 × 13.0
Presence of modified stomata	+	−
Nectary cuticle surface	striate/smooth	smooth
Phloem supply	+	−
Xylem supply	−	−
Presence of chlorophyll	+	+

#### Lateral nectaries

The lateral nectaries were semiannular, wedge-shaped with two or three shallow furrows dividing the gland into 3–4 lobes (Fig. 1b–c). The apical part of the nectary was covered with a striate cuticle (Fig. 1d–f) while in the lower parts of the secretory glands the surface of the epidermis was relatively smooth. Nectar was released through modified stomata. The nectariferous tissue bore both mature and immature solitary stomata which occurred predominantly atop the upper surface of the lobes (Fig. 1d). Stomata were placed at the same level as other epidermal cells of the nectary (Fig. 1d, e). They lacked subsidiary cells, and the cells adjoining to guard cells did not differ from other epidermal cells; thus, stomata may be considered anomocytic. Most of the stomatal apertures were wide open and were lined by well-formed cuticular ledges (Fig. 1e, f). The pores of guard cells of the modified stomata were lined by relatively thick cell wall with distinct cuticular beaks and a small substomatal space (Fig. 2e). The remnants of secreted material, presumably derived from the crystallization of the nectar, were observed on the surface of stomata and adjacent epidermal cells (Fig. 1e, f). Occasionally, a complete occlusion of the apertures of the modified stomata was also observed.

**Fig. 3:**
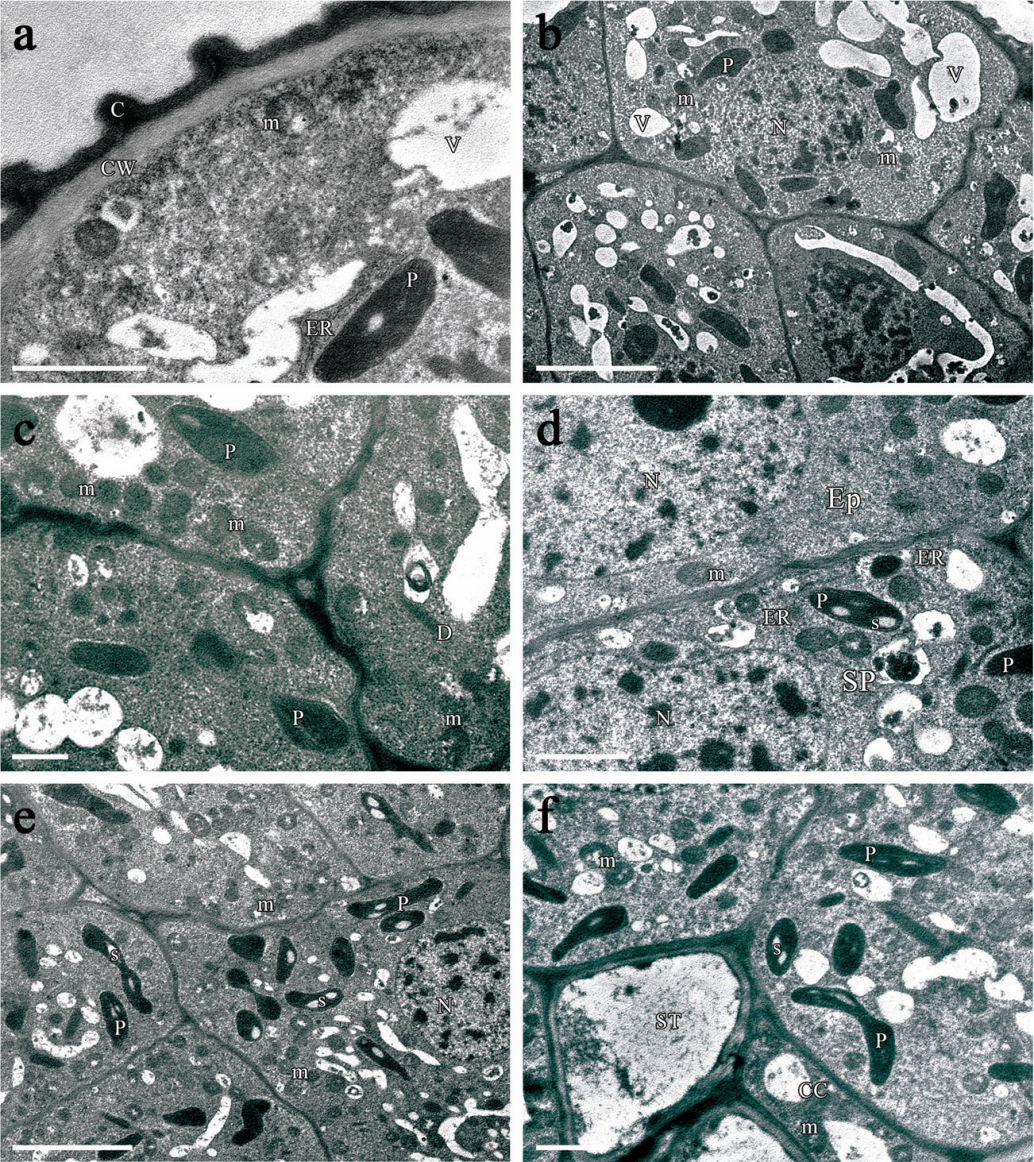
TEM micrographs of the lateral nectary of *B. orientalis*: **a** Epidermal cell with slight outward projections of the homogenous cuticle lining outer wall. In granular cytoplasm occur mitochondria, ER profiles and plastids with small, partly hydrolyzed starch grain. *Scale bar* = 2 μm. **b** Epidermal cells and subepidermal secretory parenchyma cells with large nuclei, small vacuoles and starchless plastids. *Scale bar* = 5 μm. **c** Cells of the nectar-producing parenchyma with numerous mitochondria aligned along a cell wall. *Scale bar* = 1 μm. **d** Details of epidermal and subepidermal nectary parenchyma cells. Short profiles of ER and few plastids within secretory parenchyma cells are present. *Scale bar* = 2 μm. **e** Numerous plastids with starch grains within cells located deeply in secretory parenchyma. *Scale bar* = 5 μm. **f** Sieve tube element and companion cell with mitochondria at the base of secretory parenchyma. *Scale bar* = 2 μm

Each lateral nectary consisted of a single-layered epidermis and 11–18 layers of subepidermal nectariferous parenchyma (Fig. 2a, b). The epidermis was composed of small cells (Table 3). They contained a large, centrally located nucleus (Fig. 2b, c) and were highly vacuolated (Fig. 2e, f). The relatively thick outer walls of the epidermal cells were covered by a distinct cuticle, which stained intensely with auramine O (Fig. 2f). Examination under TEM revealed slight outward projections of the cuticle overlying epidermal cells (Fig. 3a). Subepidermal nectariferous parenchyma cells had very thin cellulosic cell walls and contained intense staining cytoplasm and centrally positioned nuclei (Fig. 2b, c). Several small vacuoles, traversed by cytoplasmic strands, were present. Treatment with PAS revealed numerous starch-containing plastids which were located mostly in the deeper layers of the nectary parenchyma (Fig. 2d). Additionally, autofluorescence of the chlorophyll was observed in nectary parenchyma cells, whereas it was almost completely lacking in the epidermis (Fig. 2g). Plasmodesmatal connections between nectary parenchyma cells were observed infrequently. Intercellular spaces were located especially close to the epidermis (Fig. 2e). The nectaries were supplied by phloem alone (Table 3). Sieve tube elements of variable number and dimensions and associated companion cells were constantly detected in the nectariferous tissue. Depending on the location of phloem, discrete bundles or numerous strands of sieve tube elements were observed. However, multiple sieve tubes elements penetrated only to a depth of one to three cell layers of the nectary parenchyma (Fig. 2b, c).

Transmission electron microscopy observations revealed that secretory cells contained numerous ribosomes, resulting in highly granular appearance of the cytoplasm (Fig. 3a–f). Plastids without or with very few partly hydrolyzed starch grains were observed both in the epidermis and in the subepidermal layer of secretory parenchyma (Fig. 3a–d). In contrast, in the deeper layers of nectary parenchyma, numerous plastids with starch grains were noted (Fig. 3e, f). Dictyosomes and cisternae of endoplasmic reticulum were observed very rarely in the secretory cells (Fig. 3c, d). Numerous mitochondria were present in the nectariferous cells, being aligned along cell walls (Fig. 3c). Intercellular spaces were infrequent towards the nectary base and were relatively small (Figs. 2b and 3c, e). Radial wall plasmodesmatal connections between adjacent epidermal cells were not observed. However, plasmodesmata were infrequently present both between epidermal and subepidermal nectary parenchyma cells and between neighbouring parenchyma cells. Out of 18 sections examined here, 3 plasmodesmata were found. The sieve elements had parietal cytoplasm with very few organelles. The neighbouring companion cells contained small vacuoles, numerous mitochondria and ribosomes. In addition, wall ingrowths neither in the nectariferous nor in the companion cells were observed (Fig. 3f).

#### Median nectaries

**Fig. 4:**
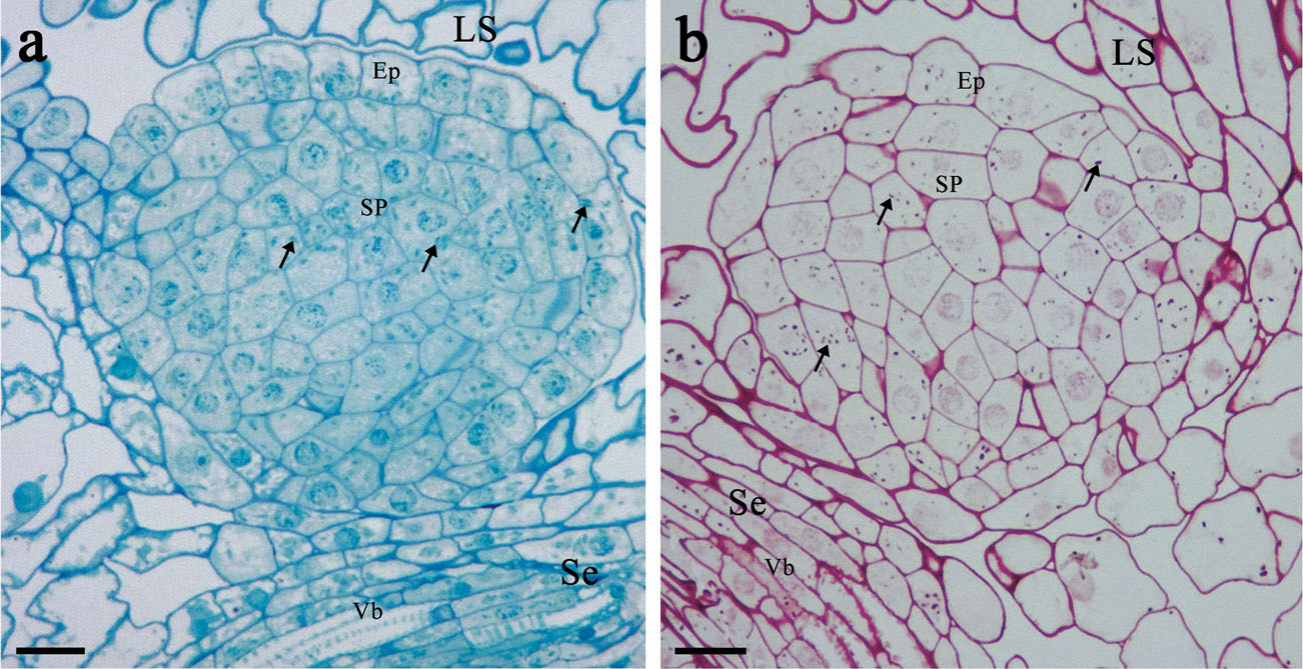
Light micrographs of the median nectary of *B. orientalis*: **a** Longitudinal section of the nectary showing highly vacuolated epidermal cells and intensely stained secretory parenchyma cells with numerous plastids (*arrows*); staining with methylene blue-Azur B. **b** Numerous starch grains (*arrows*) present in the secretory parenchyma cells; staining with PAS. *Scale bars* = 20 μm. Note the vascular bundles (Vb) that supply the sepal run at the basal part of the median nectary

Outside the bases of each pair of long stamens, median nectaries projected as prismatic outgrowths (Fig. 1b, c). They were smaller than the lateral glands. Not a single modified stoma was found on the median nectaries (*n* = 10 nectaries from five flowers of the different plants) examined (Figs. 1c and 4a, b; Table 3).

**Fig. 5:**
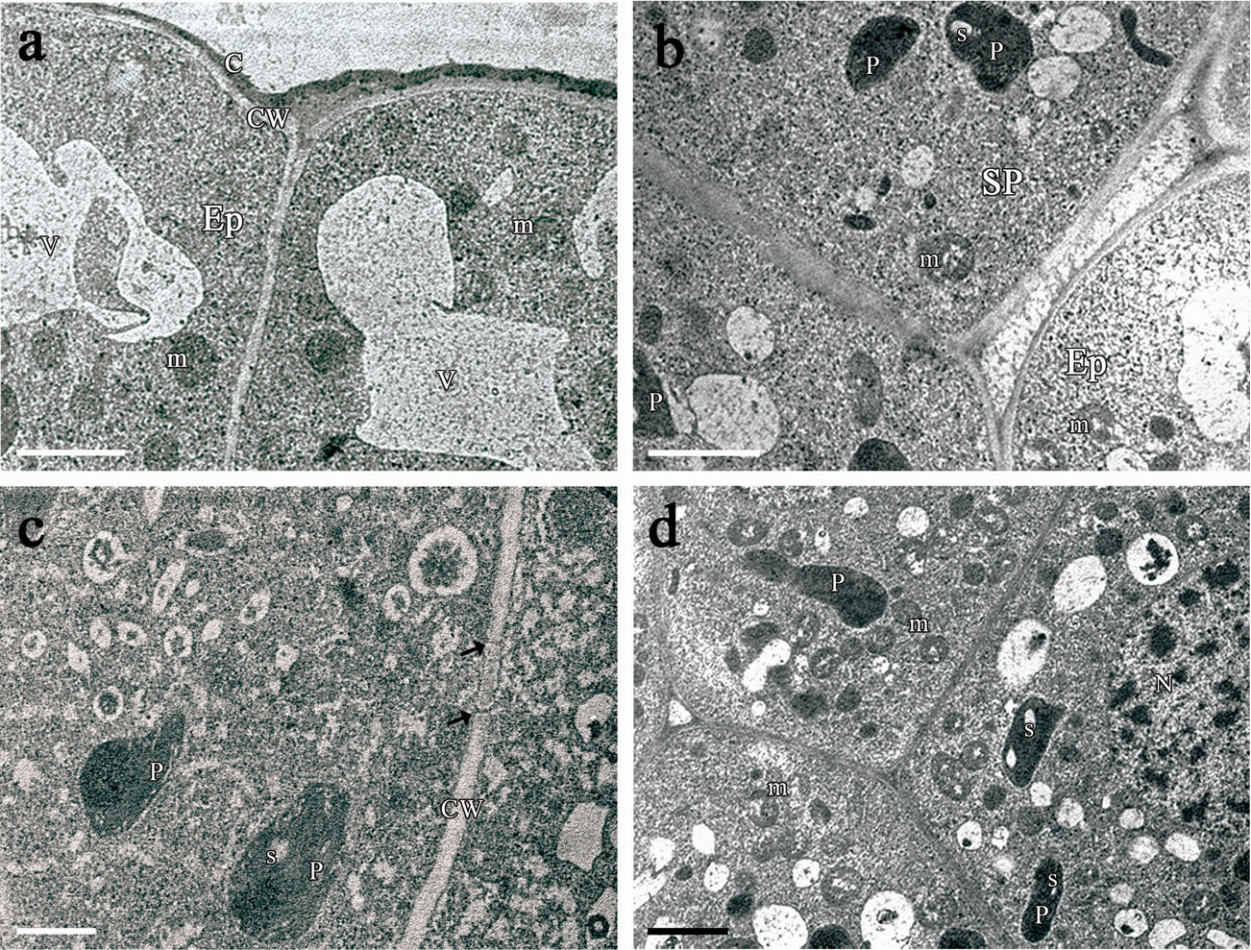
TEM micrographs of epidermal and secretory parenchyma cells of the median nectary of *B. orientalis*: **a** Epidermal cells showing granular cytoplasm with vacuoles and mitochondria. Note thick outer cell wall without projections, covered with a relatively smooth cuticle layer. **b** Epidermal cell and subepidermal secretory parenchyma cells with mitochondria, small vacuoles and plastids, note large intercellular space between epidermal and secretory parenchyma cell. **c** Secreting parenchyma cell walls with plasmodesmata (*arrows*). **d** Secretory cell with vacuoles containing fibrillar material. Also numerous mitochondria, large nucleus and starchless plastids or plastids with partly hydrolyzed starch grains. *Scale bars* = 2 μm. Abbreviations used on the figures: *C* Cuticle, *CC* Companion cell, *CW* Cell wall, *D* dictyosome, *Ep* Epidermis, *ER* Endoplasmic reticulum, *LN* lateral nectary, *LS* long stamen, *m* mitochondrion, *MN* median nectary, *N* nucleus, *Ne* nectarium, *O* ovary, *P* plastid, *Pe* petal, *s* starch grain, *Se* sepal, *SP* secretory parenchyma, *SS* short stamen, *ST* sieve tube, *V* vacuole, *Vb* vascular bundle

The median nectaries consisted of a single layer of epidermal cells and 6–10 layers of subepidermal secretory parenchyma (Fig. 4a, b). The epidermal cells and nectariferous parenchyma cells were slightly larger when compared to those in laterals (Table 3). Epidermal cells had large nuclei, several plastids with starch grains, and in contrast to nectary parenchyma cells, they were highly vacuolated (Fig. 4a). The outer cell walls were covered with a smooth cuticle (Fig. 5a). Parenchyma cells contained large nuclei and numerous plastids with starch grains (Fig. 4a, b). Their cytoplasm stained intensely with methylene blue-Azur B solution (Fig. 4a). The median nectaries were not directly vascularized; however, the vascular bundles that supply the sepals run at its basal part (Fig. 4a, b).

TEM observations revealed numerous ribosomes and mitochondria in the cytoplasm of the nectariferous cells (Fig. 5b, d). Plastids with one or two partly hydrolyzed starch grains were observed in secretory cells (Fig. 5c, d) whereas ER cisternae and dictyosomes were lacking. The small vacuoles containing osmophilic fibrillar material were numerous (Fig. 5d). Plasmodesmata were observed between secretory parenchyma cells (Fig. 5c). Intercellular spaces were small but not absent and were located in the distal region of the median nectary, especially close to the epidermis (Fig. 5b).

## Discussion

### Nectary location and structure

Flowers of *B. orientalis* possess median and lateral pair of nectaries, situated between sepals and stamens, in the region of filament bases. In general, their topography and number are similar to that reported by Appel and Al-Shehbaz (2003) and Al-Shehbaz (2010) for the genus *Bunias* and typical for members of the Brassicaceae family (e.g. Clemente Muñoz and Hernández Bermejo 1978; Davis et al. 1986; Deng and Hu 1995, Masierowska 2003). In contrast, Knuth (1908) reported that *Bunias* flowers possessed only two nectaries placed on inner sides of short stamens, which could indicate the presence only of lateral glands and a total lack of median glands. Absence of median nectaries was not confirmed in the present study.

The nectarium of *B. orientalis* is intermediate between two nectary types: the lateral and median nectary type (in which lateral and median glands are separate) and the annular nectary type (in which both nectaries are fused) sensu Deng and Hu (1995). In examined flowers, nectarial tissue of laterals partly enclosed each filament of the short stamens and a ridge of tissue connected lateral glands to median glands. Moreover, differences in the size of nectarial glands were noted; lateral nectaries were larger than median ones. Unification of nectariferous tissue and very prominent lateral glands may account for Knuth’s (1908) report of two nectaries per flower. The present results are in agreement with Appel and Al-Shehbaz (2003) and Al-Shehbaz (2010). According to these authors, lateral nectaries in *Bunias* L. are annular or semi-annular and median glands are often confluent with laterals. Connections of nectarial tissue at both lateral and median positions occur in other brassicacean species, even if the borders between individual glands can be defined, e.g. *Raphanus sativus* (Davis et al. 1998) or *Arabidopsis thaliana* (Baum et al. 2001), and unification of lateral and median glands into the annular type is considered as an evolutionary advanced trait within the Brassicaceae (Deng and Hu 1995).

The nectar-producing tissue of *B. orientalis* has a general structure comparable with that of many other brassicacean species (see Deng and Hu 1995; Davis et al. 1998 and references herein). The green glands of this species represent the type of photosynthetic nectaries consisting of epidermis, glandular tissue and phloem alone supplying lateral glands only.

The secretory cells of lateral and median nectaries of *B. orientalis* share several typical features with the nectary glands of other plant species, i.e. small size, large nuclei, small vacuoles and dense, intensely staining cytoplasm with several organelles including numerous mitochondria and ribosomes. In the secretory stage, the glandular parenchyma of both lateral and median glands contained several plastids, and in each plastid zero, one, or two starch grains were detected. Our results, strong chlorophyll autofluorescence on examination under UV light, indicate that the nectariferous tissue of *B. orientalis* can be directly involved in production of nectar carbohydrates via a photosynthesis process. According to Lüttge (2013), in sun-adapted green nectaries, photosynthesis can make a quantitatively significant contribution to the production of nectar sugar. The arrangement of floral parts in *B. orientalis* makes its nectaries only partly accessible to sunlight, and thus, their possible photosynthetic activity cannot explain the total sugar secreted in nectar. The presence of photosynthetic cells as components of floral nectaries has been described for several species (Pacini and Nepi 2007), and this phenomenon is chiefly associated with lowering the cost of reproduction and further fruit development. However, in the nectary parenchyma with chlorophyll, small quantities of starch are produced directly by photosynthesis and are stored for only few hours (Pacini et al. 2003). Recent quantitative studies have shown that starch accumulation plays only a minor role in nectar production (Ren et al. 2007; Nepi et al. 2011), and this is a characteristic of floral nectaries with a short period of secretory activity (Paiva and Machado 2008). The scarcity of starch in combination with small quantities of secreted nectar per flower indicates that floral nectaries of *B. orientalis* depend also on products of photosynthesis by other plant organs. Indeed, transport of nectar carbohydrates from other floral or vegetative parts has been demonstrated in a number of plant species (see Pacini and Nepi 2007). In lateral nectaries of *B. orientalis*, nectar carbohydrates are probably uploaded from phloem elements situated at the nectary base then transported to the secretory cells and stored temporarily as starch in plastids. Carbohydrates for nectar synthesis may also originate from vascular bundles associated with the closely located sepal bundles which may be evidenced by the presence of starch in the secretory parenchyma of median nectaries and is congruent with the observations of other species (e.g. Davis et al. 1986, 1998; Ren et al. 2007; Konarska 2011, 2015). However, the role of starch in nectar formation cannot be completely excluded for the species studied, as partly hydrolyzed starch grains have been observed in the plastids of secretory parenchyma cells.

In floral nectaries of *B. orientalis*, mitochondria occurred in great numbers whereas ER cisternae and dictyosomes, though present, were not abundant. Moreover, mitochondria were grouped along cell walls. This ultrastructural evidence suggests that an eccrine mechanism of nectar secretion, dependent on energy for active transport of sugars across cell membranes (Davis et al. 1986; Razem and Davis 1999; Wist and Davis 2006), operates here. An active transmembrane transport of nectar sugar molecules was characteristic of nectar secretory cells in another crucifer species, *Barbarea vulgaris* (Kotayeva et al. 2005). A granulocrine secretory mechanism involving reverse pinocytosis of secretory vesicles is less likely because of paucity of endoplasmic reticulum and dictyosomes in cytoplasm when compared to other nectaries (Kronestedt-Robards and Robards 1991; Fahn 2000). However, dicyosomes were present in secretory parenchyma of lateral nectaries so their involvement in a granulocrine process cannot be excluded. Similar conclusions were drawn for the floral nectaries of *Brassica napus* (Davis et al. 1986) as well as for that of *Echinacea purpurea* (Wist and Davis 2006). However, according to Matheson et al. (2006), dictyosomes are involved in the production of polysaccharides rather than the sugar components of nectar.

The receptacular nectaries of *B. orientalis* were supplied solely by phloem strands, and an inequality in phloem vascularization between lateral and median glands was observed, which is in accordance with the other Brassicaceae studied previously (see Frei 1955; Davis et al. 1986, 1998 and references herein). According to Frey-Wyssling (1955) and Fahn (1979b), there is a positive correlation between the amount of sugars in nectar and the amount of phloem elements supplying the nectaries. This correlation seems to be true for the species examined in this study, as the phloem elements are well developed in lateral glands. A remarkable feature of companion cells of phloem in *B. orientalis* nectaries is a lack of cell wall ingrowths. According to Davis et al. (1986), wall ingrowths were absent in companion cells of *B. napus* nectaries, but these cells may still function as transfer cells. They may play an important role in the unloading of phloem sap and passing pre-nectar constituents to secretory parenchyma cells and intercellular spaces next to them. The intercellular spaces are more or less conspicuous, but always are present among cells of this tissue.

Intercellular spaces located mainly in the distal part of nectaries, especially close to the epidermis, relatively thin cellulosic cell walls and the presence of plasmodesmata between nectariferous cells and between these and the epidermal cells, indicate both the apoplastic and symplastic pathway of pre-nectar and nectar movement. The models for the movement of pre-nectar along apoplast or symplast within secretory tissue and the subsequent secretion of nectar have been proposed by several researches (e.g. Kronestedt-Robards and Robards 1991; Sawidis et al. 1998; Fahn 2000; Nepi 2007; Vassilyev 2010).

Nectar exudation in lateral nectaries is achieved through nectary stomata. The nectary stomata are modified, losing the capacity to close completely (Davis and Gunning 1992). Nectar release through the nectary stomata is the most typical mode observed in many crucifers (Deng and Hu 1995; Davis et al. 1998; Masierowska 2003). The nectary stomata of *B. orientalis* are situated atop the nectary lobes at the level of the other epidermal cells. According to Nagy Tóth et al. (2000), the location of nectary stomata could be related to the adaptation of a species to its environment. The position of the stomata mainly at the level of the gland epidermal cells might indicate the occurrence of mesomorphic features in the studied species.

The apical part of lateral nectaries of *B. orientalis* was covered by a thick layer of cuticle with very distinct striation, especially in the neighbourhood of nectary stomata. The cuticle with clear and deep ornamentation favours the longer retention of nectar on the surface of the nectary and protects secretion against drying (Nagy Tóth et al. 2000). Epidermis of median glands of *B. orientalis* was smooth and completely lacked nectary stomata. Similarly, not a single modified stoma was found in the median nectaries of *S. loeselii* flowers (Davis et al. 1998). Moreover, no nectar droplet was observed on the petal claw opposite these nectaries. The differences in cuticle ornamentation between lateral and median glands of *B. orientalis* are consistent with the earlier reports on another brassicacean species *A. thaliana* (Davis 1994; Baum et al. 2001).

Under field conditions, it was not observable if, in *B. orientalis* flowers, nectar is produced by the lateral nectaries or by the medians as well. The lack of modified stomata and absence of phloem entering the median glands indicate that nectar originates from the lateral nectaries only. However, some activity of the median nectaries cannot be totally excluded as plastids with partly hydrolyzed starch grains were present within their secreting parenchyma cells. The differences in photosynthetic and secretory activity of lateral and median nectaries in *B. orientalis* flowers deserve further investigation under controlled laboratory conditions.

### Nectar production and composition and their relationship to flower visitation

Flowers of *B. orientalis* appear to be entomophilous because of their visually attractive petals, abundant pollen and presence of active nectaries. Insect pollination is important for reproductive success, i.e. seed setting, in crucifers. Even species considered as autogamous or partly autogamous benefit from insect visits; therefore, the presence of a floral reward such as nectar in their flowers is important (e.g. Williams 1978; Eisikowitch 1981; Masierowska 2013). Nectar secretion in *B. orientalis* flowers started when petals opened, with beginning of pollen shedding and lasted until petals wilted. The arrangement of floral organs makes nectar accessible for different groups of insects. In this study, the quantities of nectar produced by flowers as well as amounts of sugars secreted in nectar were relatively low. Total nectar carbohydrates production per ten *B. orientalis* flowers averaged 0.3 mg. It was about 10× and 20× lower compared to values obtained for flowers of cultivated crucifers, i.e. *Sinapis alba* (Masierowska 2003) or *Brassica napus* (Kołtowski 2002), respectively, but 3-fold higher than the amount of nectar sugar per flower in another brassicacean invasive species, *Alliaria petiolata* (Cruden et al. 1996). Mean sugar concentration of nectar, which was 28 %, was in the range reported for other crucifers (Free 1993 and references herein). These findings support the conclusion of Schürkens and Chittka (2001) about relatively low nectar productivity in *B. orientalis* flowers. According to these authors, in this species, the amount of nectar sugar flower^−1^ h^−1^ averaged 1.5 μg.

Nectar yield and quantity of sugars secreted in nectar differed substantially between vegetation seasons. Many variables can affect nectar parameters, including abiotic factors, e.g. habitat, humidity, temperature, solar radiation or CO_2_ concentration (see Nicolson and Thornburg 2007) or biotic factors such as the presence of microbial communities (Herrera et al. 2008). In our study, nectar yield and quantity of sugars were impaired during warm and dry weather conditions with air temperature approx. 1–2 °C higher and precipitation lower 50–60 % than in the long term. Furthermore, nectar volume, sugar concentration and carbohydrate composition are also related to the structural and physiological attributes of a plant itself (Shuel 1955) and its flowers (Galetto and Bernardello 2004). The nectar sugar quantity within plants of the same crucifer species can easily vary even more than 2 times (Davis et al. 1998).

The present study reveals that nectar of *B. orientalis* is hexose-dominant with overall glucose/fructose ratio greater than 1.00. This result accords with earlier reports involving analysis of the floral nectar of several species of the Brassicaceae (Percival 1961; Kevan et al. 1991; Davis et al. 1994, 1998; Farcas 2006). Furthermore, no traces of sucrose were detected in *B. orientalis* floral nectar. Percival (1961) failed to detect any sucrose in several species of the Brassicaceae, as did Davis and colleagues (1998) for *Raphanus sativus* and *Sinapis arvensis* (in median nectaries).

The flowers of *B. orientalis* contained relatively small amounts of nectar, but their energetic reward was equivalent to other species pollinated by short-tongued bees. The amount of 0.3 mg sugar per flower may be typical for short-tongued bees (Cruden and Hermann 1983). In fact, Knuth (1908) recorded small and medium-sized flies and bees as visitors to *B. orientalis* flowers. Also, Schürkens and Chittka (2001) and Denisow (2004) pointed out that the most common visitors to *B. orientalis* were short-tongued bumblebees (*Bombus terrestris* and *Bombus lapidarius*), honeybees (*Apis mellifera*) and small mining bees (*Andrena* sp.), even though pollination syndromes would categorize this flower as fly-pollinated. Since populations of *B. orientalis* include closely spaced plants displaying abundant flowers (Denisow 2011), it is not surprising that medium-sized bees and flies also foraged actively on the flowers. Floral display has been proposed to play a major role for attractiveness of other invasive plants (Vanparys et al. 2008).

In this study, significant differences between individual plants both for glucose and fructose content in nectar as well for glucose/fructose ratio were stated. However, glucose/fructose ratios always exceeded 1 and nectar was considered as hexose-dominant. The large interplant differences in total nectar-sugar production per flower within species are well known (Cruden and Hermann 1983), and nectar characteristic can be affected by various factors (as was discussed earlier) at different levels of plant organization. In the Brassicaceae, Davis et al. (1998) detected a consistent difference in nectar-carbohydrate composition of nectar provided by lateral and median nectaries located within the same flower and speculated that this disparity may have functional significance for pollination.

To conclude, floral nectaries in *B. orientalis* exhibit a number of homogenous traits to nectaries described in other representatives of the Brassicaceae. The similarities include the location and number of secretory glands in a flower, photosynthetic type of nectaries consisting of epidermis, glandular tissue and phloem alone, cuticle ornamentation, inequality in secretory activity as well as vascularisation between lateral and median glands, an eccrine mechanism of nectar secretion and nectar release through the nectary stomata. Furthermore, the sugar composition of nectar (hexose-dominant) is very common along brassicacean taxa.

The phenotypic generalization of *B. orientalis* flowers, including accessible nectaries and rewards, welcomes an array of more or less specialist insect visitors. Although the production of nectar by a single flower is low, the abundant floral display of *B. orientalis* plants as well as its occurrence in dense patches makes this species a very attractive nectar source. The impact that such mass flowering stands of *B. orientalis* make on the fitness of native plant species deserves further studies.
